# Self-Assembled Pt/MoC_x_/MWCNTs Nano Catalyst for Ethanol Electrooxidation of Fuel Cells

**DOI:** 10.3389/fchem.2022.891640

**Published:** 2022-04-12

**Authors:** Xiaochang Cao, Zhongming Qiu, Jianjun Chen, Tianyu Ai

**Affiliations:** ^1^ School of Mechanical Engineering, Dongguan University of Technology, Dongguan, China; ^2^ Dongguan JoySun New Energy Co. Ltd., Dongguan, China; ^3^ School of Materials and Metallurgy, University of Science and Technology Liaoning, Anshan, China

**Keywords:** direct ethanol fuel cell, electrocatalyst, platinum, self-assembly, molybdenum carbon

## Abstract

Direct ethanol fuel cells (DEFCs) have attracted more and more attention because of their unique advantages such as low cost and low toxicity. However, sluggish C-C bond cleavage during the ethanol electrooxidation reaction (EOR) in acidic media results in a lower energy yield and gravely hinders the commercialization of DEFCs. Therefore, it is very necessary to develop an anode catalyst with high performance, high stability and low cost to solve this problem. In this paper, Pt/MoC_x_/MWCNTs nanocomposites with different mass ratios of PtMo were obtained through a molecular self-assembly technology. The structure and morphology of Pt/MoC_x_/MWCNTs nanocomposites were characterized by several techniques such as XRD, FESEM, XPS, etc. The electrochemical performance and stability of Pt/WC_x_/MWCNTs electrocatalysts toward EOR were investigated in acid electrolytes. The results show that PtMo exists in the form of alloy. The size of Pt/MoC_x_ nanoparticles is very uniform with an average size of ∼24 nm. The Pt/MoC_0.25_/MWCNTs exhibits excellent electrocatalytic activities with an electrochemically active surface area of 37.1 m^2^ g^−1^, a peak current density of 610.4 mA mg_Pt_^−1^ and a steady-state current density of 39.8 mA mg_Pt_^−1^ after 7,200 s, suggesting that the Pt/MoC_0.25_/MWCNTs is a very promising candidate for application in EOR of DEFCs.

## Introduction

Direct ethanol fuel cells (DEFCs) have many advantages such as high power density, environmental friendliness, rapid start-up and mobility, so they are believed as the most promising high energy conversion system for practical applications in mobile devices such as automotive and portable power (Singla et al., 2017; Jiang et al., 2018; Oh et al., 2019; Wang et al., 2022). However, there are still many challenges in the energy conversion processes of DEFCs, such as the difficulty in splitting the C-C bond of ethanol and the sluggish kinetics of electrocatalytic oxidation (Du et al., 2017; Zamanzad Ghavidel et al., 2017; Zhang et al., 2018). Reasonable use of catalysts can improve the energy output efficiency and overall performance of fuel cells, the problem of incomplete oxidation of ethanol can be effectively solved in DEFCs (Corradini et al., 2015; Huang et al., 2015; Bach Delpeuch et al., 2016). At present, the most promising and active catalysts for EOR are Pt in acid media of DEFCs. However, the high price, rare reserve and its low tolerance to CO severely limit its extensive commercialization (Pech-Rodríguez et al., 2017; Yang et al., 2019; Zhu et al., 2021). Therefore, it is significant and urgent to fabricate Pt-based nanocatalysts with active C-C bond cleavage ability and enhanced CO tolerance for efficient EOR.

Transition metal carbides (TMC) with high metal conductivity, strong corrosion-resistance, high stability and rich sources have a similar catalytic activity to platinum group metals (PGMs) (Hamo et al., 2019; Zhang et al., 2019; Jiang et al., 2020; Fang et al., 2021). They have been widely concerned and applied as catalysts for fuel cells, such as WC (Hunt et al., 2014; Kelly et al., 2014; Oh et al., 2014), TaC (Myochi et al., 2018; Gao et al., 2020a), TiC (Hunt et al., 2016), Mo_2_C (Lin et al., 2017; Hassan and Ticianelli, 2018). The WC/p-CNFs composite catalyst was prepared and used in alkaline media (Oh et al., 2014). Due to the unique structure of p-CNFs and the synergistic effect between WC and p-CNFs, WC/p-CNFs composite catalyst increased the electrocatalytic performance for ethanol oxidation in DEFCs. Hunt et al. (2014) synthesized WC nanoparticles through a multi-step method. WC nanoparticles showed high electrocatalytic activity and stability under acid conditions. However, compared with Pt, TMC has lower catalytic activity for EOR, but it shows strong stability and anti-poisoning ability. Therefore, the Pt and TMC composite catalysts have been widely studied to reduce costs and further improve performance. Kelly et al. (2014) investigated ethanol electrooxidation of Pt/WC by density functional theory (DFT) and surface science experiments. The results showed that Pt/WC could oxidize ethanol to CO_2_ more effectively than Pt and improve the output power of DEFCs. Subsequently, Pt/TaC electrocatalyst was prepared by the wet impregnation method (Jiang et al., 2018). The results showed that 1.5 wt% Pt/TaC demonstrated higher activity and stability for EOR than 40 wt% Pt/C. For 1.5 wt% Pt/TaC, the Pt surface was less poisoned by EOR intermediates and had a higher CO selectivity. Besides, the DFT study showed that the binding energy of EOR intermediates on Pt (111) surface was higher than that on Pt/TaC (111) surface, which further proved that the poison tolerance of Pt/TaC was increased. Pt/Mo_2_C/C-cp catalyst was synthesized by a coprecipitation method (Li et al., 2015; Lin et al., 2017; Hassan and Ticianelli, 2018). The direct chemical bonding of Pt and MoC in Pt/Mo_2_C/C-cp catalyst significantly reduced the onset CO oxidation potential and anti-CO poisoning ability to intermediates species. The above results show that the addition of TMC to Pt nanocatalysts can diminish the overpotentials, partially facilitate the C-C bond cleavage towards CO_2_ and increase EOR activity. However, the synthesis of Pt/TMC catalyst usually requires multiple steps. TMC nanoparticles are easy to agglomerate, resulting in the reduction of specific surface area and catalytic activity.

In this work, we designed a simple molecular self-assembly technology to synthesize platinum/molybdenum carbide/multi-walled carbon nanotubes (Pt/MoC_x_/MWCNTs) as active electrocatalysts for EOR in acid media. MWCNTs have a high specific surface area and excellent electrical properties at room temperature, which are especially suitable for high-performance catalysts (Lu et al., 2012; Nie et al., 2012; Sabnis et al., 2015). Pt/MoC_x_/MWCNTs catalyst exhibits high catalytic activity and anti-CO poisoning ability. The outstanding performance of the catalyst is attributed to the complete exposure of the active sites and the synergistic effect between Pt and MoC.

## Experiment

### Materials

Hexachloroplatinic acid (H_2_PtCl_6_·6H_2_O), MWCNTs, sodium molybdate dihydrate (Na_2_MoO_3_·2H_2_O) were purchased from Shanghai Micklin Biochemical Co. Ltd. PDDA [(C_8_H_16_CIN)_n_], ethanol (CH_3_CH_2_OH), concentrated sulfuric acid (H_2_SO_4_) were purchased from AiKe reagent. Concentrated nitric acid (HNO_3_), Nafion solution (5 wt% in isopropanol and water) was purchased from Shanghai Aladdin Biochemical Technology Co. Ltd. All reagents were used in this work without further treatment.

### Synthesis of Pt/MoC_x_/MWCNTs

The samples of Pt/MoC_x_/MWCNTs were obtained by adjusting the addition amount of sodium molybdate. First, MWCNTs were treated in acid solution (90 ml H_2_SO_4_ and 30 ml HNO_3_) by stirring for 60 min and ultrasound for 60 min to form a homogeneous solution. This slurry was centrifuged and washed three times with deionized water to obtain acid-MWCNTs. Then acid-MWCNTs and PDDA were dissolved in 200 ml deionized water by ultrasonic treatment for 60 min. The mixed solution was filtered and dispersed with deionized water. Second, 0.1 mmol sodium molybdate and 0.4 mmol chloroplatinic acids were added to the above solution under magnetic stirring for 40 min. The nanopowder was obtained by freeze-drying overnight. Subsequently, the powder were sintered under Ar/H_2_ atmosphere at 1,200°C for 180 min at 2°C min^−1^. Finally, the Pt/MoC_0.25_/MWCNTs was recieved after cooling to room temperature.

### Characterization

The crystal structures were characterized by X-ray diffraction (XRD, PANalytical B.V) patterns. The data was collected from 10° to 90° at a scan speed of 15 min^−1^. The morphology and the size of Pt/MoC_x_/MWCNTs were observed by transmission electron microscopy (TEM, JEOL 2010) operating at 200 kV. The three-dimensional structure, distribution and element composition of nanoparticles on MWCNTs were observed by field emission scanning electron microscope (FESEM, JEOL JSM-6340F, 5 kV) in combination with energy-dispersive X-ray spectroscopy (EDS). The surface elemental composition and valence analysis of spherical nanoparticles were measured by X-ray photoelectron spectroscopy (XPS, PHI-5702) with a monochromatized Al Ka X-ray source (1,486.6 eV photons) and pass energy of 40 eV. The anode voltage was 15 mV with a current of 10 mA. To compensate for the effects of surface charging, all core-level spectra were referenced to the C1s hydrocarbon peak at 284.8 eV. The Raman spectrum of spherical nanoparticles was obtained by using a Renishaw RW1000 Raman spectroscope.

The electrochemical tests were performed on Autolab (PGSTAT 302N) at room temperature. The counter electrode is made of the platinum sheet with a size of 7 mm × 15 mm. For the reference electrode silver chloride (Ag/AgCl) is selected in this work. Glassy carbon electrode (GCE) with a diameter of 5 mm is used for the working electrode (WE). Catalyst inks were produced by mixing 5 mg Pt/MoC_x_/MWCNTs nanoparticles with 1 ml 0.5 wt% Nafion/isopropanol. Then, the catalyst ink was ultrasound for 20 min. Finally, 10 μl catalyst ink was dropped on the surface of GCE and dried in air. The ECSA of ETEK and Pt/MoC_x_/MWCNTs electrocatalysts was measured in a nitrogen-saturated 0.5 M H_2_SO_4_ solution at a scan rate of 50 mV s^−1^. The electrocatalytic activity for EOR was characterized by the CV measurements in a nitrogen-purged 0.5 M H_2_SO_4_ + 1.0 M ethanol solution at a scan rate of 50 mV s^−1^. Te stability was examined by CA tests at a constant potential of 0.6 V vs. Ag/AgCl.

## Results and Discussion

**FIGURE 1:**
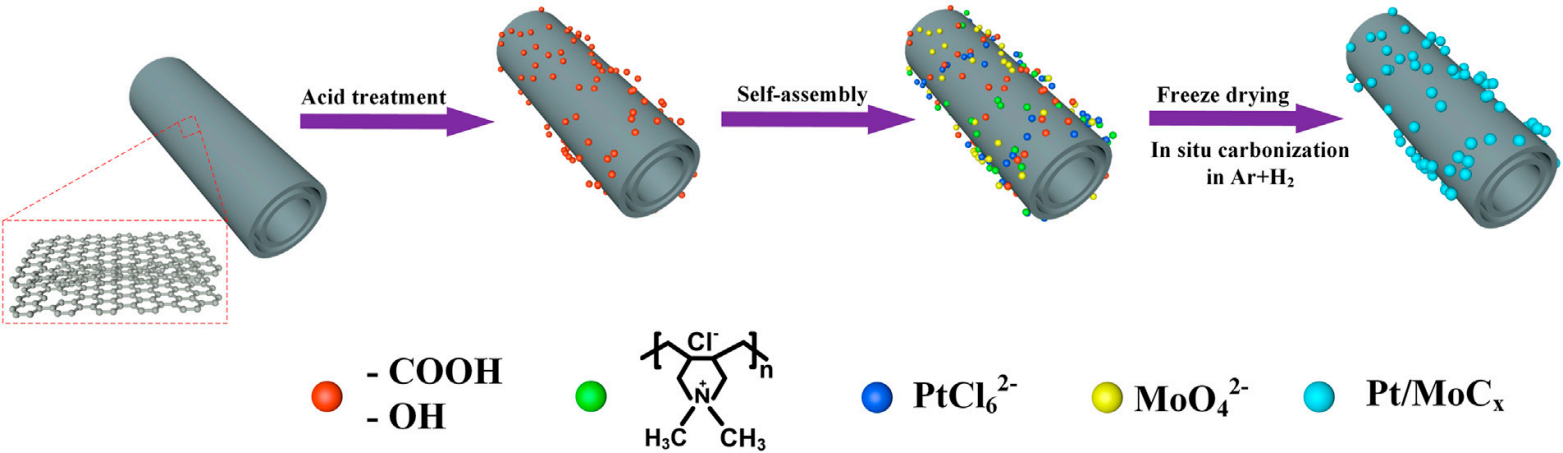
Schematic diagram for synthesizing Pt/MoC_x_/MWCNTs.

The synthesis route of Pt/MoC_x_/MWCNTs nanoparticles by molecular self-assembly is illustrated in Figure 1. Negative charges (−OH, −COOH), strong cationic polyelectrolyte PDDA, PtCl_6_^2−^ and MoO_4_^2−^ were adsorbed on MWCNTs through electrostatic adsorption to realize layer-by-layer assembly. Then, the precursors were dried under freeze-drying conditions and calcined in a weak reducing atmosphere to obtain Pt/MoC_x_/MWCNTs nanoparticles.

**FIGURE 2:**
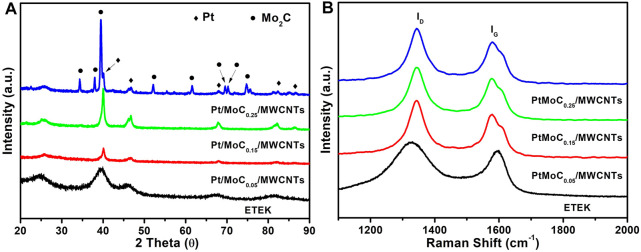
**(A)** Wide-angle XRD patterns and **(B)** Raman spectra of ETEK and Pt/MoC_x_/MWCNTs.

The XRD spectra of ETEK and Pt/MoC_x_/MWCNTs are shown in Figure 2A. The diffraction peaks at 39.44°, 46.56°, 67.98°, and 81.91° closely matched with standard values of diffraction peaks for Pt (PDF#03-065-5035) (Sabnis et al., 2015), which indicates that H_2_PtCl_6_ has been successfully reduced to Pt. However, not only the characteristic peaks of Pt but also the diffraction peaks at 34.34°, 37.98°, 39.42°, 52.14°, 61.5°, 69.56°, and 74.73° are very consistent with the standard values of Mo_2_C (PDF#00-035-0787) in the Pt/MoC_0.25_/MWCNTs (Li et al., 2019). When the content of Mo continues to increase, Mo forms PtMo alloy and Mo_2_C. The average diameters of PtMo alloy nanoparticles are calculated by the Scherrer equation, as shown in Table 1. The Scherrer constant is 0.9 and the wavelength is 1.54 Å in this case for Cu Kα radiation in the Scherrer equation. The grain sizes of Pt/MoC_0.05_/MWCNTs, Pt/MoC_0.15_/MWCNTs and Pt/MoC_0.25_/MWCNTs are about 23.72, 23.82 and 23.84 nm. Because the radius of doping Mo^4+^ ionic is large than Pt^6+^, the lattice constant of Mo doped all increase to some extent in Pt/MoC_x_/MWCNTs, compared to ETEK. These results indicate that Pt/MoC_x_/MWCNTs can be directly prepared by using the molecular self-assembly method.

**TABLE 1:** Physicochemical parameters of different sample.

Samples	Average diameter (nm)	Lattice parameters (nm)	Pt: Mo ratio (at %)
ETEK	4.36	0.389	100:0
Pt/MoC_0.05_/MWCNTs	23.72	0.391	95:5
Pt/MoC_0.15_/MWCNTs	23.82	0.391	85:15
Pt/MoC_0.25_/MWCNTs	23.84	0.391	75:25

There are two main peaks at about 1,580 cm^−1^ (G band, represents E_2g_ vibration) and 1,343 cm^−1^ (D band, represents A_1g_ vibration). The graphitization degree of MWCNTs is expressed by the relative strength of D-band (I_D_) and G-band (I_G_) (Dietrich et al., 2014). The I_G_/I_D_ ratios of ETEK, Pt/MoC_0.05_/MWCNTs, Pt/MoC_0.15_/MWCNTs and Pt/MoC_0.25_/MWCNTs are 0.9, 0.8, 0.9, and 0.9, respectively. The corresponding results indicate that the graphitization degrees of ETEK and Pt/MoC_x_/MWCNTs are similar.

**FIGURE 3:**
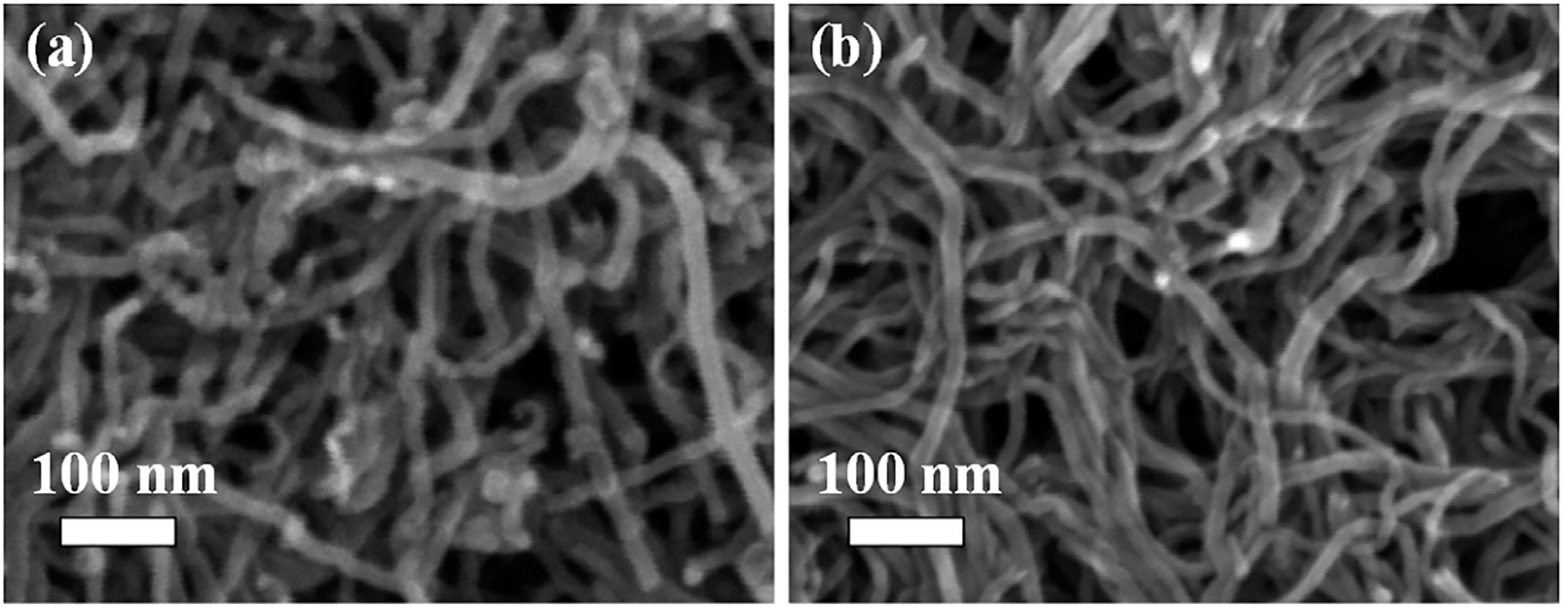
FESEM images of **(A)** MWCNTs and **(B)** acid-treated MWCNTs.

**FIGURE 4:**
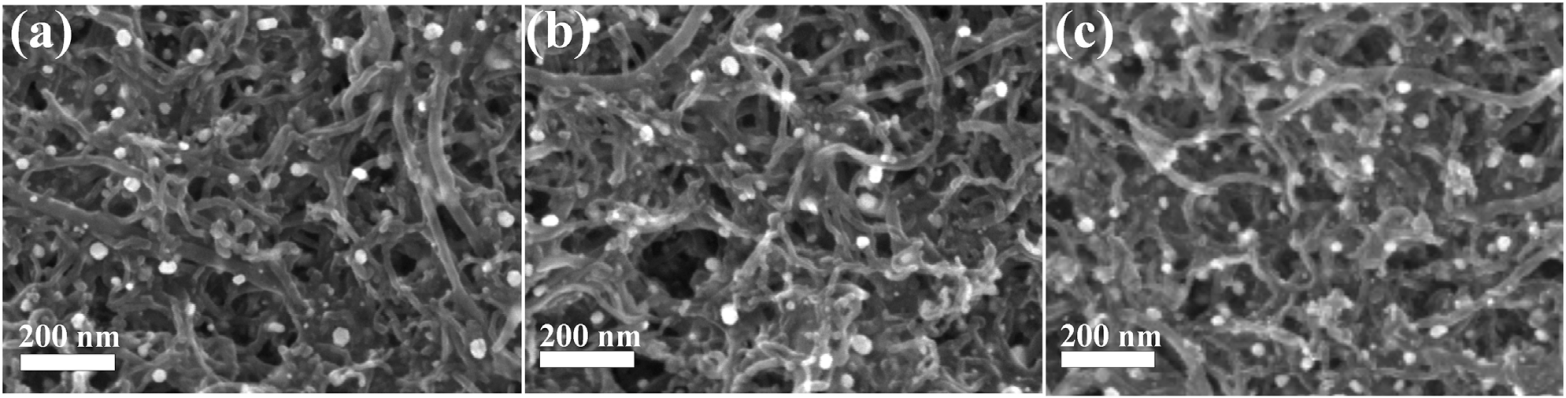
FESEM images of **(A)** Pt/MoC_0.05_/MWCNTs, **(B)** Pt/MoC_0.15_/MWCNTs and **(C)** Pt/MoC_0.25_/MWCNTs.

The SEM images of MWCNTs before and after treatment in mixed acid solution are shown in Figure 3. It can be seen that the MWCNTs have no fracture and the diameter and morphology do not change significantly after ultrasonic and mixed acid treatment. The FESEM images of Pt/MoC_0.05_/MWCNTs, Pt/MoC_0.15_/MWCNTs and Pt/MoC_0.25_/MWCNTs are shown in Figure 4. As a support material, the morphology of MWCNTs has no obvious change after composite with Mo_x_C nanoparticles, indicating that its structure has not been damaged in the synthesis process (Wang et al., 2019). Pt/Mo_x_ nanoparticles are uniformly dispersed on the surface of MWCNTs without agglomeration. The size of Pt/Mo_x_ is uniform and its particle size is about 24 nm, which is consistent with Scherrer’s calculation results. The successful preparation of Pt/Mo_x_ nanoparticles is closely related to the addition of ionic surfactant PDDA. PDDA is hydrolyzed in the precursor solution to form ion pairs. The existence of ion pairs slows down the reduction process and controls the growth rate of nanoparticles.

**FIGURE 5:**
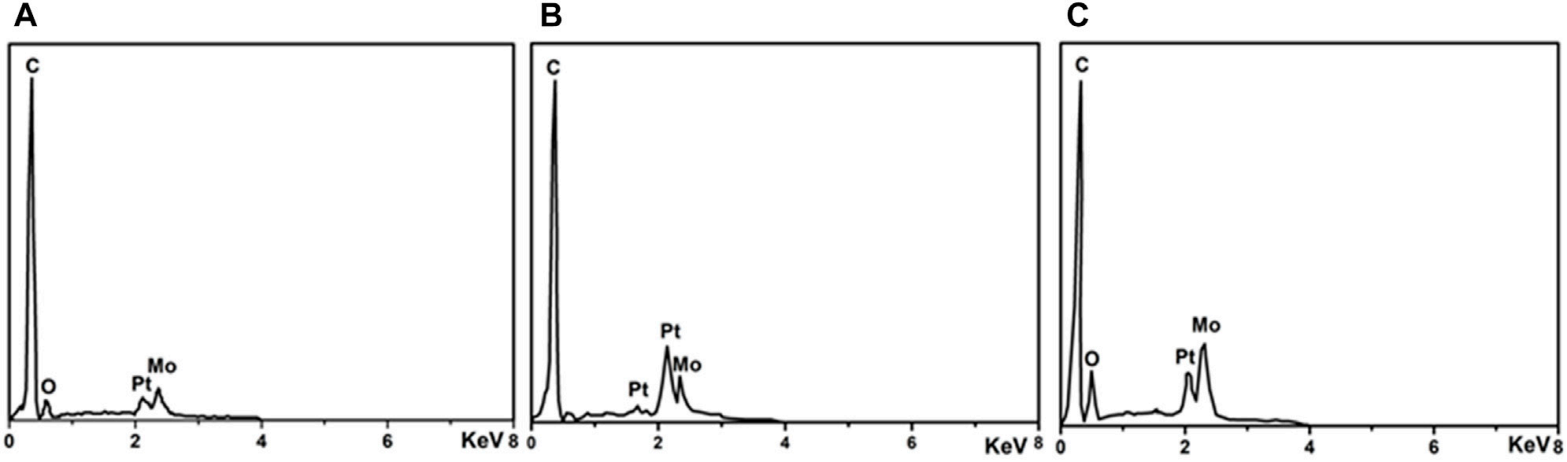
EDX analysis of **(A)** Pt/MoC_0.05_/MWCNTs, **(B)** Pt/MoC_0.15_/MWCNTs and **(C)** Pt/MoC_0.25_/MWCNTs.

**FIGURE 6:**
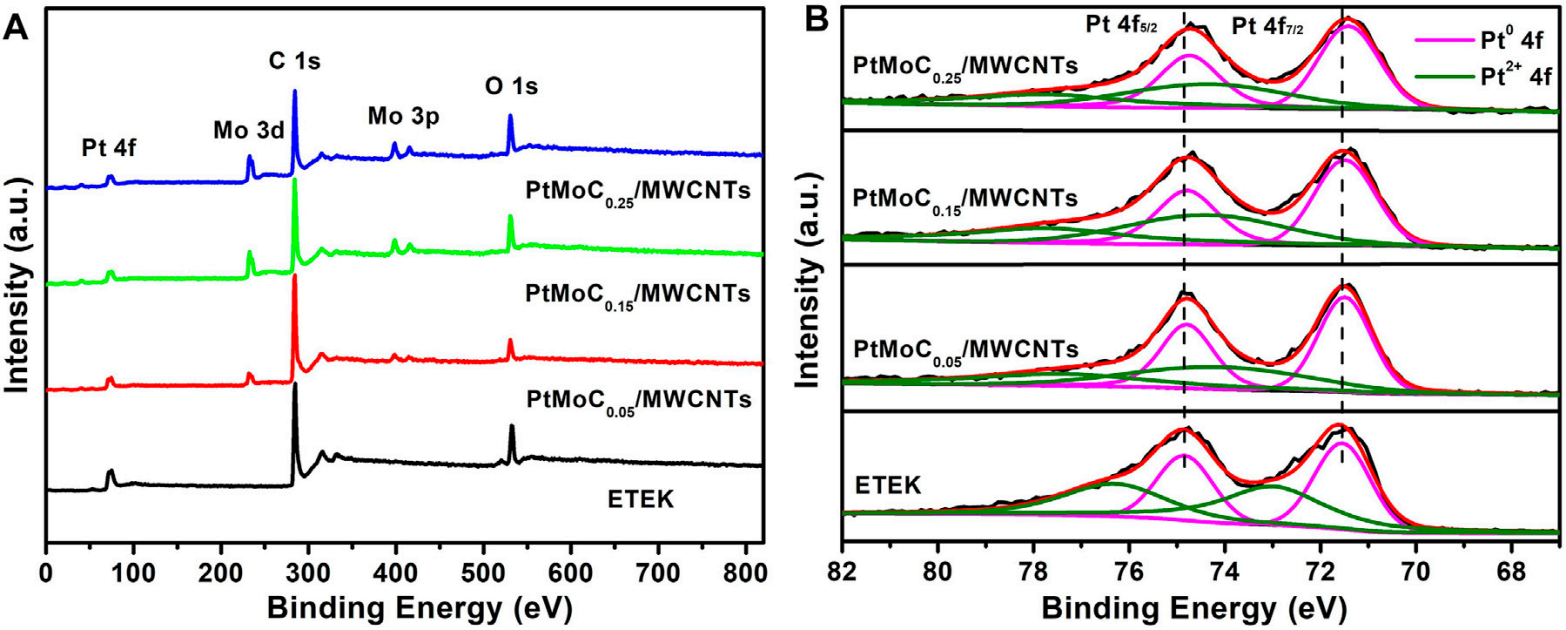
**(A)** Survey scanned XPS spectrum and **(B)** high-resolution Pt 4f spectra of ETEK, Pt/MoC_x_/MWCNTs.

Figure 5 shows the EDS results of Pt/MoC_x_/MWCNTs samples. There are characteristic peaks of C, Pt and Mo in Pt/MoC_x_/MWCNTs composites and no other element peaks. Figure 6 investigates the surface elements and chemical states of Pt/MoC_x_/MWCNTs composites by XPS. The characteristic peaks of C, Pt and Mo were observed in Pt/MoC_x_/MWCNTs composites and the intensities of characteristic peaks for Mo 3d and Mo 3p gradually increased with the increase of Mo element content as shown in Figure 6A. The surface valence state of Pt in Pt/MoC_x_/MWCNTs composites is shown in Figure 6B. The Pt 4f spectra of Pt/MoC_x_/MWCNTs show two peaks of Pt 4f_7/2_ and Pt 4f_5/2_ and can be further divided into two doublet peaks, which associates with metal Pt and Pt oxide. It is worth noting that, compared with ETEK, the bond energy of Pt 4f in Pt/MoC_x_/MWCNTs shifts slightly to the negative phase and the peak binding energy is shown in Table 2. The shift of bond energy is caused by the electronegativity of Pt, which may cause the more charges transformation from Mo to Pt. The decrease of Pt bond energy will weaken the adsorption energy of Pt and CO_ads_, promote the removal of CO_ads_ and promote the breaking of C-H (Lu et al., 2016; Gao et al., 2020b).

**TABLE 2:** Binding energies of XPS spectra of Pt in ETEK and Pt/MoC_x_/MWCNTs.

Sample	Species			
	Pt^0^ 4f_7/2_	Pt^2+^ 4f_7/2_	Pt^0^ 4f_5/2_	Pt^2+^ 4f_5/2_
ETEK	71.55	73.05	74.85	76.35
Pt/MoC_0.05_/MWCNTs	71.47	74.2	74.77	77.5
Pt/MoC_0.15_/MWCNTs	71.45	74.39	74.75	77.69
Pt/MoC_0.25_/MWCNTs	71.42	74.47	74.72	77.77

**FIGURE 7:**
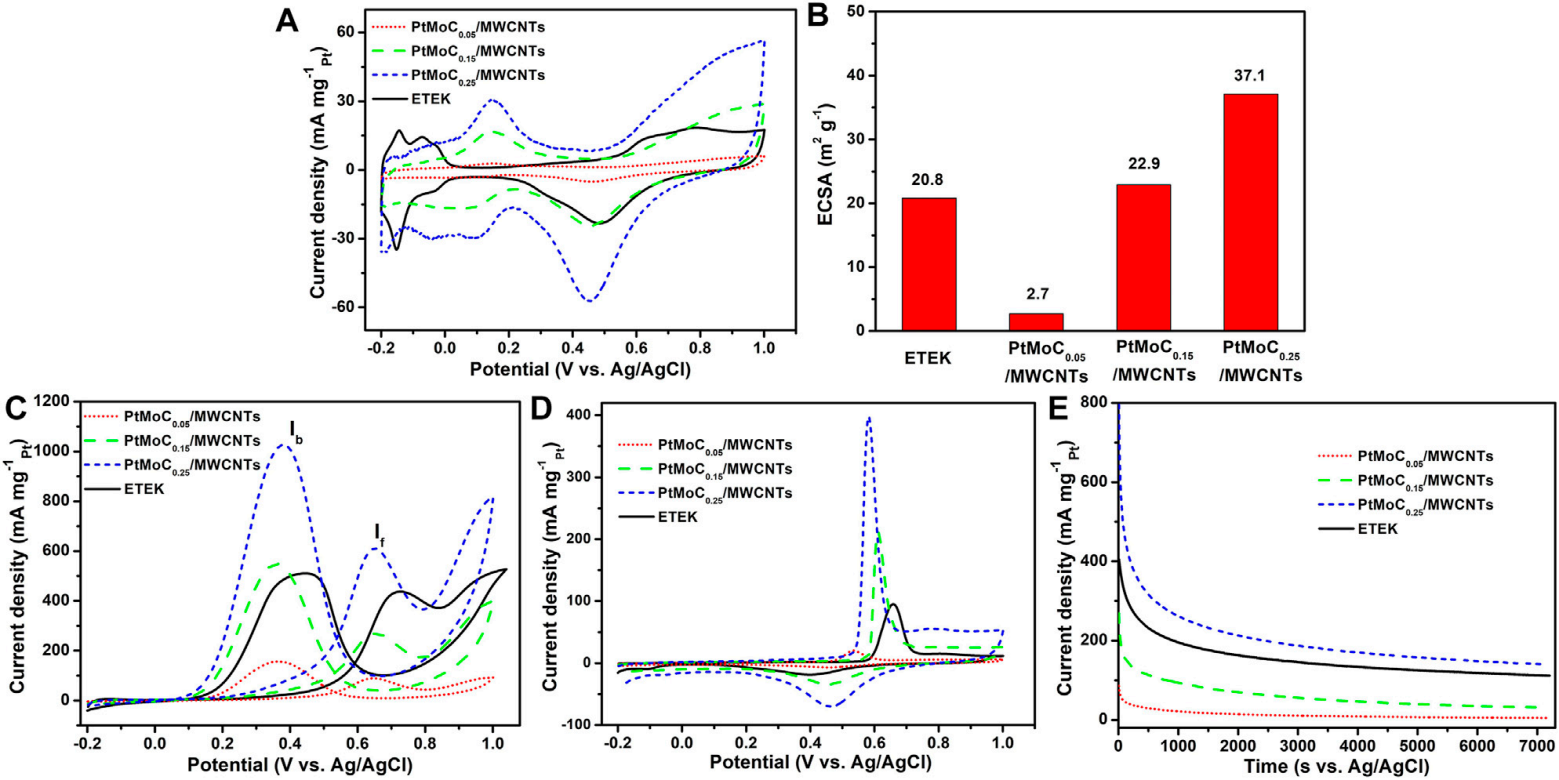
CV of the catalysts **(A)** in a N_2_-saturated 0.5 M H_2_SO_4_ solution, **(B)** ECSA, **(C)** in a N_2_-saturated 0.5 M H_2_SO_4_ + 1 M CH_3_CH_2_OH solution, **(D)** CO stripping voltammograms in a 0.5 M H_2_SO_4_ solution at 25°C with a scan rate of 50 mV s^−1^ and **(E)** CA curves of the catalysts in a N_2_-saturated 0.5 M H_2_SO_4_ + 1 M CH_3_CH_2_OH solution at 25°C.

The electrocatalytic activity of Pt/MoC_x_/MWCNTs composites catalyst in acid medium is shown in Figure 7. Figure 7A shows the cyclic voltammetry (CV) curves of ETEK, Pt/MoC_0.05_/MWCNTs, Pt/MoC_0.15_/MWCNTs and Pt/MoC_0.25_/MWCNTs catalysts in a N_2_-saturated 0.5 M H_2_SO_4_ solution. In Figure 7B, the electrochemically active surface area (ECSA) can be obtained from the hydrogen adsorption/desorption region in a 0.5 M H_2_SO_4_ solution. The specific value of ECSA for the Pt/MoC_0.25_/MWCNTs is 37.1 m^2^ g^−1^, which is slightly higher than that commercial TETK electrocatalyst (20.8 m^2^ g^−1^). Due to the uniform distribution of Pt/MoC nanoparticles and the electronic structure change caused by the introduction of Mo, the ECSA area is increasing. These results indicate that more Pt active sites on Pt/MoC_0.25_/MWCNTs are exposed for EOR (Dai et al., 2020).

In Figure 7C, the electrocatalytic activities of Pt/MoC_x_/MWCNTs and ETEK for EOR were tested in a N_2_-saturated 0.5 H_2_SO_4_ with 1.0 M CH_3_CH_2_OH solution. During the EOR forward scanning of Pt/MoC_x_/MWCNTs electrocatalysts, there is one peak at 0.65 V, which is caused by the fracture of the C-C or C-O bond (ethanol oxidations to acetaldehyde, acetic acid and CO_2_). During the reverse scanning, the peak current appeared at 0.38 V related to the oxidation of intermediates from ethanol dissociative adsorption (e.g., CO). It is known that the excessive accumulation of intermediates leads to catalyst poisoning. Therefore, the current density ratio between peak f and b (I_f_/I_b_) has been used to evaluate the anti-poison capability of electrocatalysts. According to the calculation results, the surface of Pt/MoC_x_/MWCNTs shows higher anti-CO poisoning ability than ETEK. As can be seen from Figure 7C, the peak current densities of Pt/MoC_0.25_/MWCNTs is 610.4 mA mg_Pt_^−1^, which is about 1.4 times that of ETEK (449.2 mA mg_Pt_^−1^). Due to the synergistic effect between Pt and MoC and the promotion of MoC in the adsorption of reaction and the desorption of products, the EOR activity of the composite catalyst is significantly increased (Robinson et al., 2016; Xiao et al., 2021). EOR performance and the synthesis method of the current work were compared to relevant reports, as shown in Table 3.

**TABLE 3:** Comparisons of the EOR performance for Pt based catalysts in recently published papers.

Refs.	Catalyst	ECSA (m^2^ g^−1^_Pt_)	Electrolyte	Mass activity (mA mg^−1^_Pt_)	Methods
13	Pt/BC	52.7	0.5 M H_2_SO_4_ +	770	Solvent heating method
			1.0 M CH_3_CH_2_OH		
30	Rh@Pt d-CNCs	34.65	0.1 M HClO_4_ +	860	Solvent heating method
			0.2 M CH_3_CH_2_OH		
34	PtCo@N-GNS-3	—	0.5 M H_2_SO_4_ +	196	Hydrolysis-pyrolysis method
			0.5 M CH_3_CH_2_OH		
35	Pt-AuSnO_x_	44.1	0.5 M H_2_SO_4_ +	305	Improved impregnation method
			1.0 M CH_3_CH_2_OH		
37	Pt/α-PtO_x_/WO_3_	151.6	0.1 M NaOH +	2,760	One-pot solvothermal method
			1.0 M CH_3_CH_2_OH		
This work	Pt/MoC_x_/MWCNTs	37.1	0.5 M H_2_SO_4_ +	610.4	Molecular self-assembly method
			1.0 M CH_3_CH_2_OH		

The anti-poisoning of Pt/MoC_x_/MWCNTs catalyst is a very important parameter in practical application. The CO stripping voltammetry curve was tested in 0.5 M H_2_SO_4_, as shown in Figure 7D. The onset potentials of CO for ETEK, Pt/MoC_0.05_/MWCNTs, Pt/MoC_0.15_/MWCNTs and Pt/MoC_0.25_/MWCNTs were 0.58, 0.45, 0.57, and 0.48 V, respectively. The Pt/MoC_x_/MWCNTs catalyst can oxidize CO at low potential, which makes CO easier to desorb from the surface of nanoparticles, releases more active sites and improves the performance of Pt/MoC_x_/MWCNTs catalyst. In addition, the peak voltage of Pt/MoC_0.25_/MWCNTs is about 80 mV lower than ETEK, indicating that Pt/MoC_0.25_/MWCNTs catalyst has higher CO oxidation activity.

In Figure 7E, the stability of Pt/MoC_x_/MWCNTs catalyst is tested by the chronoamperometric (CA) method at a constant potential of 0.6 V for 7,200 s. As shown in the current-time curves, the initial current values of ETEK, Pt/MoC_0.05_/MWCNTs, Pt/MoC_0.15_/MWCNTs and Pt/MoC_0.25_/MWCNTs catalysts are 404.6, 85.8, 268.9 and 820.2 mA mg_Pt_^−1^, respectively. The polarization current of all catalysts decreases sharply within 200 s, which is caused by the poisoning of Pt/MoC_x_ and the reduction of catalytic active sites caused by the intermediates during the electrooxidation of ethanol (Mao et al., 2017). In the following time, the current gradually reaches a plateau due to the established balance between the adsorption and oxidation of the intermediates. After 7,200 s, the current of Pt/MoC_0.25_/MWCNTs still reached 139.8 mA mg_Pt_^−1^, which is higher than that of ETEK. MoC can significantly improve the stability of Pt/MoC_0.25_/MWCNTs catalyst and reduce the adsorption of intermediate products on the catalyst surface. The higher stability current achieved on Pt/MoC_0.25_/MWCNTs compared to ETEK, together with the results from the above CV tests, confirms the best EOR performance of Pt/MoC_0.25_/MWCNTs.

## Conclusion

In summary, Pt/MoC_x_/MWCNTs nanocomposites were successfully synthesized by the molecular self-assembly technology. The structural characterization shows that Pt/MoC_x_ nanoparticles are evenly dispersed and anchored on MWCNTs. The structure of MWCNTs is not significantly damaged during the synthesis process. The electrochemical measurement results show that Pt/MoC_0.25_/MWCNTs electrocatalyst has the highest catalytic activity and the best stability. The significant improvement of electrochemical performance is attributed to the introduction of MoC, which changes the electronic structure of Pt/MoC_x_, provides more active sites for the EOR, enhancing the electrocatalytic activity. The synergistic effect between Pt and MoC provides more active sites for intermediates and improves the catalytic activity. And the MoC interface is beneficial to the adsorption of reaction products and the desorption of intermediate products, which improves the catalytic activity of the composite catalyst. The newly developed self-assembly technique has a great deal of potential for synthesizing Pt/carbide nanocomposite electrocatalysts and the as-prepared Pt/MoC_0.25_/MWCNTs demonstrates a promising prospect as anode catalyst for applying in DEFCs.

## Data Availability

The original contributions presented in the study are included in the article/Supplementary Material, further inquiries can be directed to the corresponding authors.
